# IL-1*β* and TNF*α* Promote Monocyte Viability through the Induction of GM-CSF Expression by Rheumatoid Arthritis Synovial Fibroblasts

**DOI:** 10.1155/2014/241840

**Published:** 2014-11-17

**Authors:** Christelle Darrieutort-Laffite, Marie-Astrid Boutet, Mathias Chatelais, Régis Brion, Frédéric Blanchard, Dominique Heymann, Benoit Le Goff

**Affiliations:** ^1^INSERM, UMR 957, 44035 Nantes, France; ^2^Rheumatology Unit, Nantes University Hospital, 44093 Nantes, France; ^3^Université de Nantes, Nantes Atlantique Universités, Laboratoire de Physiopathologie de la Résorption Osseuse et Thérapie des Tumeurs Osseuses Primitives, Faculté de Médecine, 44035 Nantes, France

## Abstract

*Background*. Macrophages and synovial fibroblasts (SF) are two major cells implicated in the pathogenesis of rheumatoid arthritis (RA). SF could be a source of cytokines and growth factors driving macrophages survival and activation. Here, we studied the effect of SF on monocyte viability and phenotype. *Methods*. SF were isolated from synovial tissue of RA patients and CD14+ cells were isolated from peripheral blood of healthy donors. SF conditioned media were collected after 24 hours of culture with or without stimulation with TNF*α* or IL-1*β*. Macrophages polarisation was studied by flow cytometry. *Results*. Conditioned medium from SF significantly increased monocytes viability by 60% compared to CD14+ cells cultured in medium alone (*P* < 0.001). This effect was enhanced using conditioned media from IL-1*β* and TNF*α* stimulated SF. GM-CSF but not M-CSF nor IL34 blocking antibodies was able to significantly decrease monocyte viability by 30% when added to the conditioned media from IL-1*β* and TNF*α* stimulated SF (*P* < 0.001). Finally, monocyte cultured in presence of SF conditioned media did not exhibit a specific M1 or M2 phenotype. *Conclusion*. Overall, rheumatoid arthritis synovial fibroblasts stimulated with proinflammatory cytokines (IL-1*β* and TNF*α*) promote monocyte viability via GM-CSF but do not induce a specific macrophage polarization.

## 1. Introduction

Rheumatoid arthritis is an autoimmune disease characterized by a chronic inflammation of the synovial membrane that leads to joint destruction. The inflammatory and resident cells present in the synovial tissue play a major role in the pathogenesis of the disease through the production of a wide range of proinflammatory cytokines and growth factors. Synovial fibroblasts (SF) and macrophages are two cell types composing the lining layer of the normal synovial tissue. They are also the main cells found in the inflamed synovium of RA patients. In RA, SF acquire a pseudotumoral phenotype characterized by a higher proliferation rate, invasiveness, and a resistance to apoptosis leading to hypertrophy of the lining layer [1, 2]. These cells play a key role by producing cytokines (IL-6 and receptor activator for nuclear factor *κ* B ligand (RANKL)) that perpetuate inflammation and induce bone destruction and metalloproteases that contribute to cartilage damage. Besides their contribution to cartilage and bone damage, SF seem to play a crucial role in the homing, growth, or function of other cell types, such as inflammatory cells [3]. For instance, SF are a source of chemokines and also of hematopoietic growth factors such as monocyte chemoattractant protein 1 (MCP-1), macrophage colony stimulating factor (M-CSF), IL-34, or granulocyte-macrophage colony-stimulating factor (GM-CSF) that can support monocyte migration and macrophage activation in the synovium.

Macrophages are also among the major cells involved in the pathogenesis of inflammatory arthritis. These cells are abundant in the inflamed synovial tissue and their number in the synovial sublining layer is correlated with disease activity and response to treatment [4, 5]. Their importance is also underlined by the efficacy of therapies targeting macrophage-derived cytokines (TNF*α* or IL-1*β*) in RA [6]. These cells can be derived from circulating monocytes which are recruited in the synovial tissue or from the differentiation of macrophage precursors already present in the joint. It has been shown that the main factors of monocytes/macrophages survival such as M-CSF, IL-34, or GM-CSF are expressed in the synovial fluid and membrane of RA patients [7–9]. The involvement of these factors in the inflammation and destruction of the joint has been demonstrated in animal models of RA. Thus, op/op mice lacking functional M-CSF are protected against antigen induced arthritis [10]. Furthermore, GM-CSF has been shown to exacerbate arthritic disease in animals, whilst GM-CSF deficient mice are protected from collagen-induced arthritis [11, 12]. These studies demonstrate that M-CSF and GM-CSF can exacerbate the inflammatory response and support the role of monocytes/macrophages in the pathogenesis of inflammatory arthritis.

It is increasingly clear that macrophages are a heterogeneous population and that they should be classified according to their phenotype and function [13]. Macrophages can be subdivided in two groups: classically activated macrophages (M1) which are proinflammatory via the secretion of cytokines such as TNF*α*, IL-1*β*, and IL-12 and alternatively activated macrophages (M2) which have anti-inflammatory functions (secretion of IL-10, IL-1RA, and transforming growth factor-*β*) and promote tissue repair [14]. The phenotype of synovial macrophages is heterogeneous as suggested by the concomitant expression of markers M1 and M2 [15]. The macrophage polarization may vary depending on the disease activity or the treatment such as glucocorticoids that are known to promote M2 polarization [16]. It has also been hypothesized that an imbalance between pro- and anti-inflammatory macrophages could be involved in the chronicity of inflammation.

SF and macrophages are in close contact in the synovium. As the SF are sources of cytokines and growth factors, it has been hypothesized that these cells may regulate the survival and activation of monocytes/macrophages in the synovial tissue. However, it is not known what factors (M-CSF, IL-34, or GM-CSF) are involved in the promotion of monocytes survival in the rheumatoid synovium. This question is important because new therapeutic strategies targeting M-CSF and GM-CSF are in development. Moreover, it is unclear whether SF are able to influence macrophage polarization. In this work, we have shown that RA SF can promote monocyte viability through the secretion of GM-CSF and not of M-CSF or IL-34. IL-1*β* was the main cytokine inducing the production of GM-CSF by SF. Finally, SF could not induce specific M1 or M2 phenotype.

## 2. Materials and Methods

### 2.1. Human Samples

All patients enrolled have given their formal consent. The study was approved by the local ethics committee and by the French Research Ministry (N°2008-402) in accordance with the Declaration of Helsinki.

#### 2.1.1. CD14+ Monocytes Isolation

Blood samples were obtained from the “Etablissement Français du Sang”. For CD14+ monocytes, peripheral blood mononuclear cells from 10 different donors were isolated by centrifugation over Ficoll gradient (Sigma-Aldrich, USA). CD14+ cells were magnetically labeled with CD14 microbeads and positively selected by MACS technology (Miltenyi Biotec, Germany). CD14+ cells were CD3− by flow cytometry (purity ≥ 95%) and were frozen prior to further experiments.

#### 2.1.2. Synovial Fibroblasts and Synovial Fluids

Synovial biopsies were obtained surgically at the time of joint replacement surgery or joint synovectomy from rheumatoid arthritis patients. Overall, biopsies from 9 different patients were used for our experiments. SF were obtained from synovial tissue after incubation in collagenase A (1 mg/mL) (Sigma-Aldrich) for 2 hours. After filtration with a 70 *μ*m cell strainer, cells were cultured in RPMI 1640 medium (Lonza, Belgium) supplemented with 10% of fetal bovine serum (ThermoScientific, USA) and 1% of antibiotics (penicillin/Streptomycin (Lonza)). Nonadherent cells were removed by washing with Dulbecco's phosphate buffered saline (DPBS) (Lonza) at 24 h. Analysis by flow cytometry showed expression of CD90 by more than 90% of the isolated cells. Cells were used between passage 3 and 8.

Synovial fluids were obtained from the Rheumatology Department of the Nantes University Hospital. Cells were removed by centrifugation (3600 rpm, 15 minutes) and fluids were stored immediately at −80°C. Synovial fluids were obtain from 14 osteoarthritis (OA) and 31 RA patients during a flare of the disease. Patients were suffering from RA for 188 months (± 140). Rheumatoid factor and anti-CCP antibodies were positive in 71% and 76% of patients, respectively, and 62% of patients were positive for both. Sixty-seven percent of these patients had bone erosions.

### 2.2. Synovial Fibroblasts Conditioned Media

SF conditioned media were generated from RA SF or cultured in RPMI with 2% of FBS for 12 hours and treated or not (CT) with 25 ng/mL of TNF*α* or IL-1*β* (R&D Systems) for 24 hours. At the end of the stimulation, the conditioned media were centrifugated (5 minutes, 1600 rpm) to remove cells and debris, aliquoted, and stored at −80°C after that. Conditioned media from OA patients were also generated without stimulation by cytokine.

### 2.3. RNA Isolation and Real-Time PCR

RA SF total RNA was extracted using Trizol reagent (Invitrogen, France). First-strand cDNA was synthesized from 1 *μ*g total RNA using the Maxima H Minus First Strand cDNA Synthesis Kit (ThermoScientific). Real-time PCR was performed using SYBRGreen Supermix (Bio-Rad, France). Quantitative PCRs (qPCRs) were carried out on a CFX96 Real-Time PCR Detection System (Bio-Rad). Analyses were performed using human hypoxanthine-guanine phosphoribosyltransferase (HPRT) as invariant control.

### 2.4. WST-1 Viability Assay

After thawing, CD14+ monocytes were counted and resuspended in *α*MEM medium supplemented with 20% FBS. In a 96-well plate, we added 50 *μ*L of cells in each well (45,000 cells/well) and then 50 *μ*L of M-CSF, IL-34, and GM-CSF (all from R&D systems) diluted in 2% RPMI to obtain a 25, 50, and 10 ng/mL final concentration, respectively, or 50 *μ*L of RA SF conditioned media. Medium (50 *μ*L; 2% FBS) without any cytokine was used as control condition (CT). After 3 days, cell viability was assessed by WST-1 cell proliferation Reagent (Roche, France) by adding 10 *μ*L per well. The formazan dye obtained from WST-1 cleavage by mitochondrial dehydrogenase was read by a microplate reader Wallac 1420 Victor 2 (Perkin Elmer, USA) between 420 and 480 nm after incubation for 4 hours at 37°C. Antibodies used to identify cytokines implicated in monocyte viability were as follows: anti M-CSF (R&D Systems), anti-IL-34 (Diaclone, INSERM UMR957), and anti-GM-CSF (R&D Systems) at 2, 10, and 5 *μ*g/mL, respectively. We have previously tested different antibody concentrations to determine the most appropriate concentrations.

### 2.5. Quantification of Cytokine Levels in Synovial Fluids and Conditioned Media

GM-CSF levels were measured in RA SF conditioned media by ELISA assay (Human GM-CSF DuoSet ELISA development kit, R&D Systems). In synovial fluids, GM-CSF, IL-1*β*, and TNF*α* levels were measured using the Luminex technology (Bio-Plex Pro Assays from Bio-Rad) and M-CSF levels using ELISA Assay (Human M-CSF Duoset, R&D Systems).

### 2.6. Flow Cytometry

To determine the phenotype of differentiated cells obtained in the presence of RA SF conditioned media, we used flow cytometry. CD14+ monocytes were cultured 4 days in *α*MEM supplemented with 10% FBS alone or with IFN*γ* (50 ng/mL; M1) or IL-4 (50 ng/mL; M2a) or IL-10 (50 ng/mL; M2c) or RA SF conditioned media diluted at 1/2. The cells were collected using StemPro Accutase (Life Technologies) washed with DPBS and incubated for 1 hour with the following antibodies: anti-CD14/Brilliant Violet 605, anti-CD16/Brilliant Violet 421, anti-CD64/Alexa Fluor 488, anti-CD163/Alexa Fluor 647, and anti-CD200R/Phycoerythrine (PE) (all from BioLegend, USA). Cells were analyzed with a BD LSR II flow cytometer (BD Biosciences) using BD FACSDiva Software (BD Biosciences). Values are expressed as the ratio of mean fluorescence intensity (MFI) of the marker on stimulated cells over MFI of unstimulated cells (CD14+ monocytes cultured 4 days in *α*MEM supplemented with 10% FBS only).

### 2.7. Statistics

We used a Kruskall-Wallis test for multigroup comparison. Mann-Whitney test was then used to compare each group. Correlation between cytokines concentration in synovial fluids was studied using the nonparametric Spearman rank order test. *P* < 0.05 was considered statistically significant.

## 3. Results

### 3.1. Synovial Conditioned Media Increase Monocyte Viability

**Figure 1:**
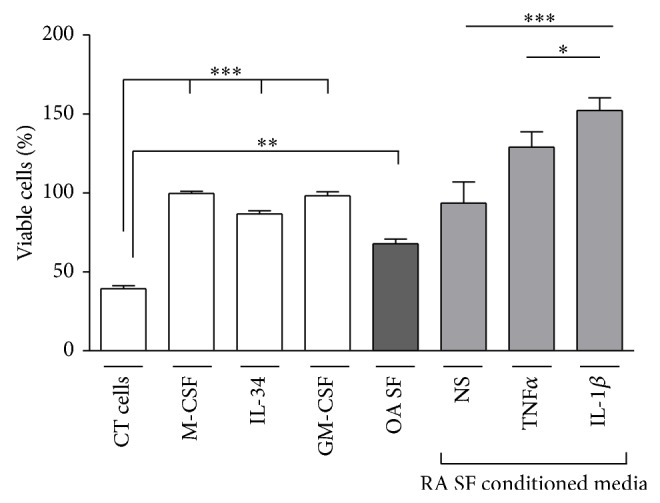
*SF conditioned media promote monocyte viability*. CD14+ cells were cultured 3 days in presence of medium alone (control (CT) cells), M-CSF, IL-34, GM-CSF, or conditioned media from RA SF stimulated or not (NS) with TNF*α* or IL-1*β* for 24 hours and from not stimulated OA SF. Monocyte viability was evaluated by colorimetric assay (WST-1). Results are given as the percentage of viability induced by M-CSF (100%) (*n* = 9 patients for RA and 2 patients for OA). ^*^ *P* < 0.05; ^**^ *P* < 0.001; ^***^ *P* < 0.0001.

First, we investigated whether soluble factors produced by SF could promote monocyte viability. CD14+ cells isolated from healthy donors were cultured for 3 days in presence of conditioned media from RA SF. Cell viability in each condition of conditioned media was evaluated by colorimetric assay (WST-1) and compared to the viability induced by M-CSF, IL-34, or GM-CSF. Results are expressed in percentage of viability induced by M-CSF (100%). As shown in Figure 1, monocyte viability was significantly increased by conditioned media compared to control cells. This effect was equivalent to that observed with M-CSF, GM-CSF, or IL-34 when using conditioned medium from nonstimulated SF. In contrast, this effect was stronger when using conditioned media from SF prestimulated 24 hours with IL-1*β* or TNF*α*. In these conditions, monocyte viability was significantly increased compared to M-CSF alone (+29% (*P* = 0.05) and +52% (*P* = 0.004) for TNF*α* and IL-1*β* conditioned media, resp.). OA SF conditioned medium induced a significant increase in monocyte viability compared to CD14 alone (*P* < 0.001) but this effect was weaker than the one induced by M-CSF or RA SF conditioned media (*n* = 2).

### 3.2. Synovial Fibroblasts Express the Main Monocyte Survival Factors M-CSF, IL-34, and GM-CSF and This Expression Is Increased by TNF*α* or IL-1*β* Stimulation

**Figure 2:**
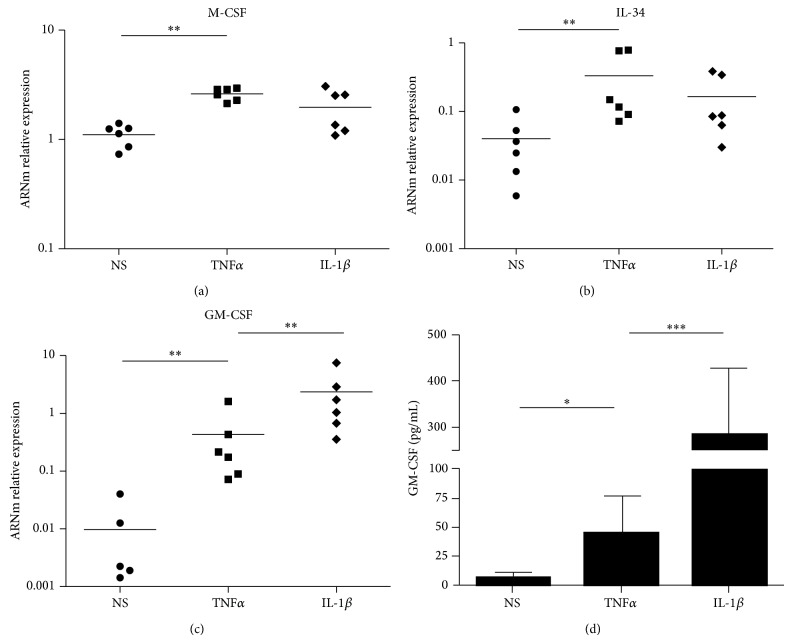
*Effect of IL-1*β* and TNF*α* on M-CSF, IL-34, and GM-CSF expression in RA SF*. SF were stimulated by IL-1*β* (25 ng/mL) or TNF*α* (25 ng/mL) for 24 hours. (a)–(c) RNA was harvested and a qPCR for M-CSF, IL-34, and GM-CSF expression was performed. Results are given as the relative expression using hHPRT gene as housekeeping gene. The bars represent the mean and each plots one biological replicate (*n* = 6 patients). (d) GM-CSF concentration (pg/mL) was assessed by ELISA. Results are given as the mean (± SEM). ^*^ *P* < 0.05; ^**^ *P* < 0.001; ^***^ *P* < 0.0001.

As SF conditioned media were able to promote monocyte survival, we then studied the expression of the main monocyte survival factors by SF (M-CSF, IL-34, and GM-CSF) and their regulation by the proinflammatory cytokines IL-1*β* and TNF*α*. After 24 hours of culture with or without TNF*α* (25 ng/mL) or IL-1*β* (25 ng/mL), synoviocytes RNA was harvested and gene expression studied by qPCR. As shown in Figure 2, unstimulated SF expressed M-CSF and IL-34 whereas GM-CSF was weakly expressed, with no expression in some SF. TNF*α* stimulation significantly increased M-CSF, IL-34, and GM-CSF expression by SF. In contrast, IL-1*β* stimulation had no significant effect on M-CSF and IL-34 expression but was able to increase GM-CSF expression by 450 (±233)-fold, compared to unstimulated cells. We confirmed these results at the protein level by ELISA assay: IL-1*β* stimulation significantly increased GM-CSF concentration (285 pg/mL (±141)) compared to TNF*α* stimulation (45 pg/mL (±30)) and to unstimulated cells (7 pg/mL (±3) (*P* = 0.03 and *P* = 0.0006 for TNF*α* and IL-1*β*, resp.)).

### 3.3. Anti-GM-CSF Antibodies but Not M-CSF or IL-34 Antibodies Inhibit the Monocyte Viability Induced by Conditioned Media from SF Prestimulated with IL-1*β* and TNF*α*

**Figure 3:**
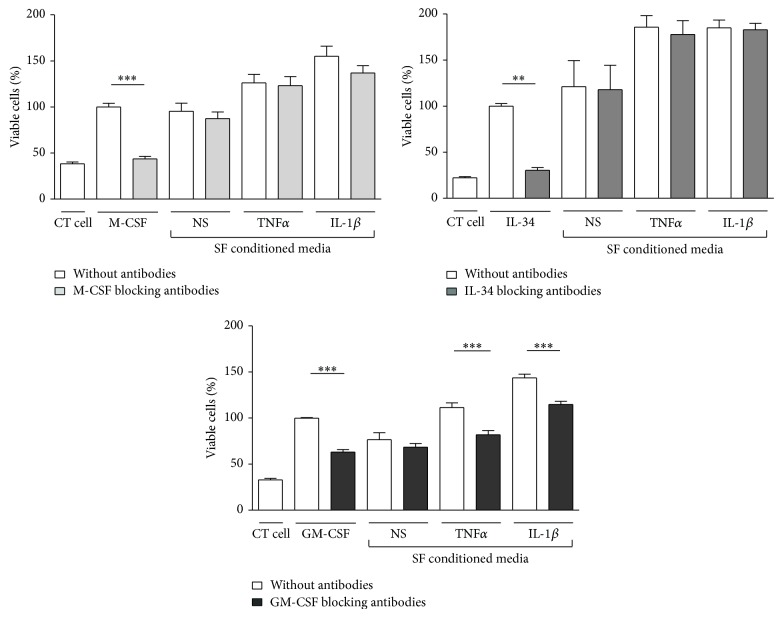
*Neutralisation of GM-CSF but not M-CSF nor IL-34 reduces monocyte viability induced by IL-1*β* and TNF*α* prestimulated SF conditioned media*. CD14+ cells were cultured 3 days in presence of M-CSF (25 ng/mL), IL-34 (50 ng/mL), GM-CSF (10 ng/mL), or conditioned media from SF prestimulated or not (NS) with TNF*α* or IL-1*β* for 24 hours. Blocking antibodies against M-CSF, IL-34, and GM-CSF, at 2, 10, and 5 *μ*g/mL, respectively, were added at the same time. Results are given as the mean percentage of viability (± SEM) considering M-CSF as control (100%) (*n* = 3–5 patients). ^*^ *P* < 0.05; ^**^ *P* < 0.001; ^***^ *P* < 0.0001.

We then wanted to identify which factors of M-CSF, IL-34, or GM-CSF were involved in maintaining monocyte viability in SF conditioned media. Freshly isolated CD14+ cells were cultured 3 days with SF conditioned media in the presence of M-CSF, IL-34, or GM-CSF blocking antibodies. As shown in Figure 3, no antibody was able to inhibit the effect of unstimulated SF conditioned media. Only GM-CSF blocking antibodies inhibited 30% of the effect of conditioned media from IL-1*β* or TNF*α* prestimulated SF. In addition, blocking antibodies targeting the IL-6 family (anti-gp130 and anti-IL-6) had no biological effect (data not shown). Thus, GM-CSF is one of the cytokines involved in the effect of SF on monocyte survival but only after cell stimulation by proinflammatory cytokines.

### 3.4. Macrophages Induced by Synovial Fibroblasts Conditioned Media Do Not Exhibit a Specific Phenotype

**Figure 4:**
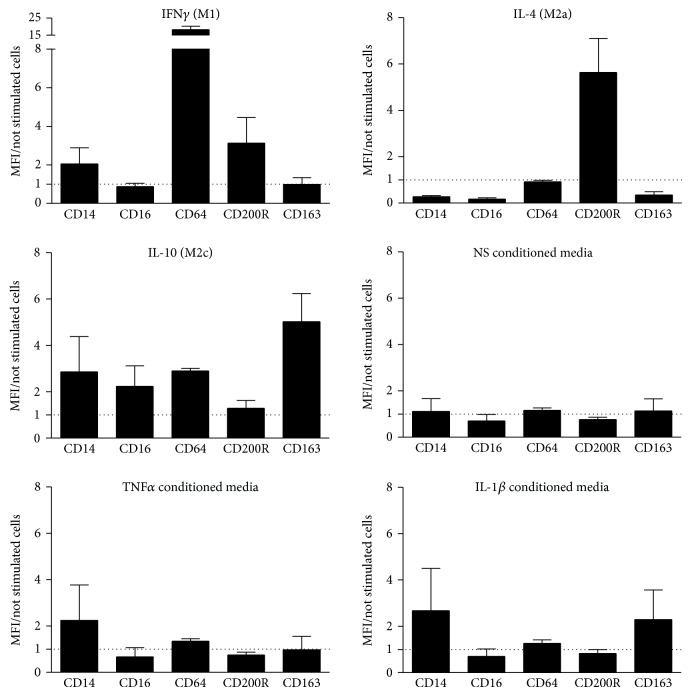
*SF conditioned media do not regulate the expression of typical M1 or M2 surface markers*. CD14+ monocytes were cultured 4 days in presence of medium with 10% FBS without (not stimulated cells) or with IFN*γ* (50 ng/mL; M1) or IL-4 (50 ng/mL; M2a) or IL-10 (50 ng/mL; M2c) or RA SF conditioned media. FACS analysis of the cell surface markers for classical (CD14), nonclassical (CD16), M1 (CD64), M2a (CD200R), and M2c (CD163) macrophages was thus performed. Values are expressed as the ratio of the mean fluorescence intensity (MFI) of the marker on the stimulated cells over MFI of unstimulated cells. Bars represent the mean (± SEM) of 3 independent experiments.

Macrophages can be subdivided in 2 specific groups: classically activated macrophages (M1) that are proinflammatory and alternatively activated macrophages (M2) which have an anti-inflammatory and tissue repair function. To determine the phenotype of macrophages induced by SF conditioned media, we performed a FACS analysis of the cell surface markers for classical (CD14), nonclassical (CD16), M1 (CD64), M2a (CD200R), and M2c (CD163) macrophages (Figure 4). Results are expressed as the ratio of the mean fluorescence intensity (MFI) of the marker over the MFI of unstimulated cells. As expected, stimulation with INF-gamma, IL-4, and IL-10 increased CD64 (18.3 ± 3.4-fold), CD200R (5.6 ± 2.9-fold), and CD163 (5 ± 2.4-fold) expression, respectively. In contrast, we did not find any regulation of the cell surface markers on the cells differentiated with unstimulated SF conditioned media. A slight but not significant increase in CD14 was observed in monocytes cultured with TNF*α* and IL-1*β* stimulated SF conditioned media and in CD163 in those cultured with IL-1*β* stimulated SF conditioned media. Overall, our data show that although SF conditioned media increase monocyte viability and proliferation, they cannot induce M1 or M2 macrophages markers.

### 3.5. GM-CSF Concentration Is Increased in Synovial Fluids from RA Patients and Is Correlated with TNF*α* and IL-1*β* Concentrations

**Figure 5:**
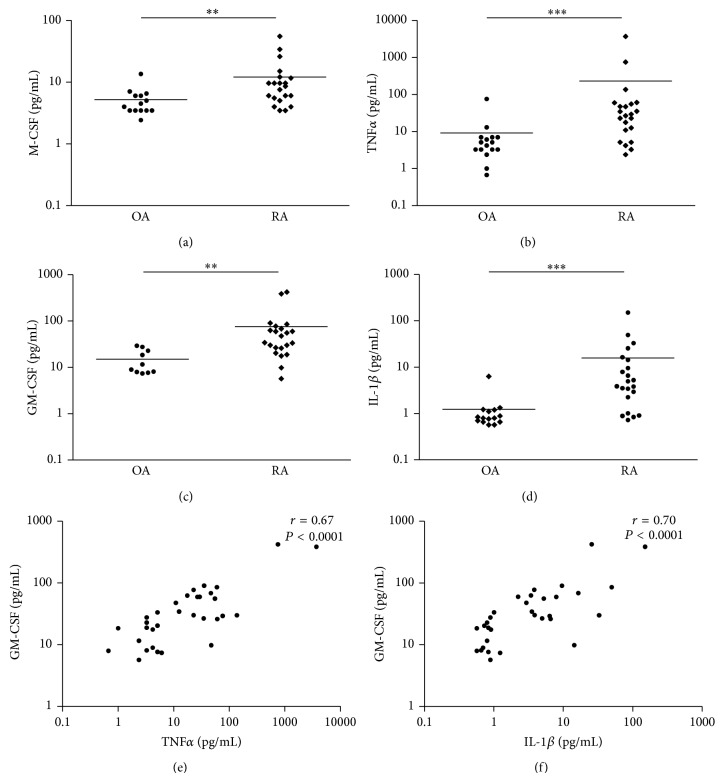
*MCSF, GM-CSF, TNF*α*, and IL-1*β* concentrations are increased in the synovial fluid of RA patients*. (a)–(d) MCSF, GM-CSF, TNF*α*, and IL1 concentration was assessed in synovial fluid of RA (*n* = 21) and OA (*n* = 14) patients using the Luminex technology. Each dot represents one patient and the bar indicates the mean concentration. ^**^ *P* < 0.001; ^***^ *P* < 0.0001. (e)-(f) Scatter plot showing the correlation between TNF*α*, IL-1*β*, and GM-CSF concentration. *r* = correlation coefficient measured using the nonparametric Spearman rank order test.

Cytokines present in the synovial microenvironment are also found in the synovial fluid of patients with inflammatory arthritis. We have already shown that IL-34 was elevated in RA synovial fluids compared to osteoarthritis (OA) [7]. Thus, we next assessed concentrations of M-CSF, GM-CSF, IL-1*β*, and TNF*α* in the synovial fluids (Figure 5). We found a significant increase in the levels of all these cytokines in RA compared to OA. Mean M-CSF concentrations were 5.2 pg/mL (± 0.75) and 12 pg/mL (± 2.7) (*P* = 0.0067) whereas GM-CSF mean concentrations were 14 (± 2.7) pg/mL and 75.8 (± 23.3) pg/mL (*P* = 0.0011) in the OA and RA group, respectively. As expected, TNF*α* concentration was higher in RA than in OA synovial fluids (9.2 (± 4.5) pg/mL versus 231 (± 168)) and IL-1*β* (1.2 (± 0.37) pg/mL versus 15.8 (± 6.2) pg/mL for OA and RA patients, resp.). A positive and significant correlation was found between GM-CSF concentration and these 2 cytokines concentrations (*r* = 0.67 and *r* = 0.70 with TNF*α* and IL-1*β*, resp.; *P* < 0.0001). These results show that the three main factors of monocyte viability are increased in synovial fluids from RA patients compared to OA patients. A positive correlation exists between IL-1*β* and TNF*α* concentrations and GM-CSF level. Thus, in inflammatory conditions, GM-CSF concentration is increased in the joint and can play a role in RA patients on monocyte viability.

## 4. Discussion

Macrophages are key cells involved in the physiopathology of RA. These cells are abundant in the synovial membrane and are the main source of proinflammatory cytokines such as TNF*α* or IL-1*β*. The mechanisms leading to the accumulation of macrophages in the synovial tissue are not entirely clear. Using labelled autologous monocytes, Thurlings et al. showed that, in RA, only a small number of peripheral monocytes migrate into the synovial compartment, indicating that the turnover of synovial macrophages from circulating monocytes is slow [17]. Moreover, studies have shown a decrease in macrophage apoptosis in the synovial tissue [5]. This indicates that there is an increased viability of macrophage precursors at the site of inflammation and that targeting factors promoting this viability could be a therapeutic approach in RA. In this study, we showed that RA SF are able to promote monocyte viability and that GM-CSF, mainly induced by IL-1*β* and TNF*α*, contributes to this effect.

Synovial membrane is composed of resident cells such as SF that are able to interact with inflammatory cells present in the tissue. For instance, it has been shown that SF can promote B cells viability* in vitro* through IL-15 expression [18]. These cells can also promote CD4 T cells and neutrophils survival but not their proliferation [19]. Coculture of SF with U937 cells promotes cartilage degradation through an increase in the expression of MMPs by SF, showing that the inflammatory cells can in turn modulate SF activity [20]. Here, we showed that SF conditioned media can promote monocyte viability with the same efficacy as M-CSF, IL-34, or GM-CSF. Moreover, prestimulation of the synoviocytes for 24 hours with TNF*α* or IL-1*β* further enhances this effect. Interestingly, conditioned media from IL-1*β* stimulated SF induced the maximal effect, showing a significant increase in monocyte viability compared to M-CSF alone. As macrophages are one of the main sources of IL-1*β* in the synovial membrane, we can speculate that they might in turn activate SF to produce factors influencing their own viability.

To identify which factors were involved in the effect of SF on monocytes, we next assess the expression by SF of the three main monocyte survival factors (IL34, M-CSF, and GM-CSF) and inhibit their effect using blocking antibodies. M-CSF was the first of the hematopoietic growth factors to be isolated [21]. It has already been shown that SF were able to produce M-CSF, this expression being increased by dexamethasone or retinoid and decreased by indomethacin [8, 22, 23]. It has thus been hypothesized that M-CSF was the main SF derived factor involved in increasing monocyte survival in RA. However, to our knowledge, no study has given direct proof of this effect. Here, we confirmed that M-CSF is expressed by synoviocytes and regulated by proinflammatory cytokines. However, we have shown that M-CSF blocking antibodies were unable to prevent the proviability effect of SF conditioned media. In line with this result, Dickerson et al. recently showed that SF conditioned media can promote osteoclastogenesis but the addition of M-CSF in the culture medium was required to observe this effect [24]. Thus, if M-CSF is expressed by SF, its biological activity needs to be further explored.

IL-34 is a newly discovered cytokine that can substitute for M-CSF to induce monocytes survival and osteoclastogenesis [25]. We were the first to demonstrate that RA SF produce IL-34 and that this expression was increased by TNF*α* stimulation [7]. Here, we showed that blocking IL-34 had no effect on monocyte viability induced by SF conditioned media. Hwang et al recently studied the role of IL-34 produced by SF on osteoclastogenesis and human PBMC's migration [26]. They showed that addition of a blocking antibody against IL-34 to SF conditioned media reduced the migration of human mononuclear cells in a dose-dependent manner. Moreover, the addition of anti-IL-34 antibodies also significantly reduced osteoclasts formation. However, these authors used PBMC's as a source of osteoclasts. As IL-34 has been shown to induce IL-17 expression by PBMC's [27], it remains unclear whether the observed effect is related to a direct effect of IL-34 or to factors produced by other cells such as lymphocytes.

In this study, we identified GM-CSF as one of the growth factors involved in monocyte viability induced by SF conditioned media. Interestingly, this effect was only observed using conditioned media from IL-1*β* or TNF*α* prestimulated SF. This could be explained by the low level of expression of GM-CSF by SF in the absence of cytokine stimulation. We found that IL-1*β* was more potent than TNF*α* to induce GM-CSF production. These results are in line with previous studies that show the importance of IL-1*β* in the regulation of growth factor expression in SF [28, 29]. This effect is not limited to this type of cells and has been described, for instance, with human lung fibroblasts. Their production of GM-CSF is also increased in response to IL-1*β* stimulation [30].* In vivo*, IL-1*β*-induced arthritis is less severe in GM-CSF−/− mice showing that a part of IL-1*β* effect is mediated by GM-CSF [31]. Finally, GM-CSF effect is not limited to the monocyte lineage as it is also able to increase neutrophils and SF survival [32, 33]. However, even if significant, we showed that anti-GM-CSF antibody inhibits monocyte viability to an extent of about 30% showing that other factors able to modulate monocyte survival are present in the ST conditioned medium. Nevertheless, these results confirm that GM-CSF could be an interesting target in the treatment of RA. This is in line with the promising effect of mavrilimumab, a monoclonal antibody targeting the alpha subunit of the GM-CSFR, actually in clinical phase II studies [34].

Macrophages observed in the inflamed synovial tissue of RA patients represent a heterogeneous population composed of both pro- and anti-inflammatory cell subtypes [35]. Their specific polarization status might change during the course of the disease and must be determinant in the phenotype and severity of the disease. Moreover, high plasticity is one of the characteristics of these cell populations. This plasticity is regulated by the balance of pro- and anti-inflammatory signals [36]. Ambarus et al. studied the phenotype of macrophages by immunohistochemistry in synovial tissue of RA and SPA patients [15]. They confirm this heterogeneity as a majority of cells coexpressed M1 and M2 markers. Using classical and validated markers of M1 and M2 macrophages, we studied* in vitro* the effects of SF conditioned media on macrophage polarization by FACS. Although classical markers of M1, M2a, and M2c were induced by Interferon gamma, IL4, and IL10, respectively, no specific markers were upregulated in macrophages differentiated in the presence of SF conditioned media. Conditioned media represents a mix of different cytokines and growth factors that act at the same time on the cells. GM-CSF differentiates macrophages in M1 phenotype [37]. On the other hand, M-CSF and IL-34 are known to polarize macrophages towards immunosuppressive ones [38]. Interestingly, Foucher et al. showed that GM-CSF prevents the generation of immunosuppressive macrophages induced by IL-34 [39]. This shows that final polarization of cells is the result of complex interactions between cytokines and growth factors. If SF cannot induce a specific phenotype, these cells could simply act to maintain the viability and increase the half-life of immature monocytes, the polarization being the consequence of factors secreted by other cell types present in the inflammatory microenvironment (B cells, T cells, and mast cells).

Overall, SF produces factors that enhance monocyte viability. Interestingly, in absence of TNF*α* or IL-1*β* stimulation, the classical monocytes survival factors (M-CSF, GM-CSF, or IL-34) do not appear to play a significant role in this effect. This shows that unknown cytokines and growth factors acting on monocytes might be involved and are still to be discovered. Moreover, targeting one cytokine might not be sufficient because SF conditioned media is a mix of cytokines and growth factors that interact together to control monocyte viability. We also showed that IL-1*β* is one of the main drivers of monocyte survival through the induction of GM-CSF production by SF. It is known that IL-1*β* is one of the main cytokines involved in crystal induced arthritis and that macrophages also play a central role in these diseases. Considering this, targeting GM-CSF could also be promising in gout or calcium pyrophosphate deposition disease (CPPD). Finally, if SF conditioned media did not induce any of the classical macrophages phenotype, these cells could promote the viability of immature monocytes within the synovial membrane which could then be targeted by other cytokines produced by inflammatory cells of the microenvironment.
